# Detection of Proteome Diversity Resulted from Alternative Splicing is Limited by Trypsin Cleavage Specificity*[Fn FN2]

**DOI:** 10.1074/mcp.RA117.000155

**Published:** 2017-12-08

**Authors:** Xiaojing Wang, Simona G. Codreanu, Bo Wen, Kai Li, Matthew C. Chambers, Daniel C. Liebler, Bing Zhang

**Affiliations:** From the ‡Lester and Sue Smith Breast Center, Baylor College of Medicine, Houston, Texas 77030;; §Department of Molecular and Human Genetics, Baylor College of Medicine, Houston, Texas 77030;; ¶Department of Biochemistry, Vanderbilt University School of Medicine, Nashville, Tennessee 37232;; ‖BGI-Shenzhen, Shenzhen, Guangdong 518083, China;; **Jim Ayers Institute for Precancer Detection and Diagnosis, Vanderbilt-Ingram Cancer Center, Nashville, Tennessee 37232

## Abstract

Alternative splicing dramatically increases transcriptome complexity but its contribution to proteome diversity remains controversial. Exon-exon junction spanning peptides provide direct evidence for the translation of specific splice isoforms and are critical for delineating protein isoform complexity. Here we found that junction-spanning peptides are underrepresented in publicly available mass spectrometry-based shotgun proteomics data sets. Further analysis showed that evolutionarily conserved preferential nucleotide usage at exon boundaries increases the occurrence of lysine- and arginine-coding triplets at the end of exons. Because both lysine and arginine residues are cleavage sites of trypsin, the nearly exclusive use of trypsin as the protein digestion enzyme in shotgun proteomic analyses hinders the detection of junction-spanning peptides. To study the impact of enzyme selection on splice junction detectability, we performed *in-silico* digestion of the human proteome using six proteases. The six enzymes created a total of 161,125 detectable junctions, and only 1,029 were common across all enzyme digestions. Chymotrypsin digestion provided the largest number of detectable junctions. Our experimental results further showed that combination of a chymotrypsin-based human proteome analysis with a trypsin-based analysis increased detection of junction-spanning peptides by 37% over the trypsin-only analysis and identified over a thousand junctions that were undetectable in fully tryptic digests. Our study demonstrates that detection of proteome diversity resulted from alternative splicing is limited by trypsin cleavage specificity, and that complementary digestion schemes will be essential to comprehensively analyze the translation of alternative splicing isoforms.

RNA sequencing (RNA-Seq) studies have highlighted a key role of alternative splicing in increasing transcriptome complexity. It has been shown that 92–94% of human genes undergo alternative splicing, and about 86% have a minor isoform frequency of 15% or more (1). However, the contribution of alternative splicing to proteomic complexity remains controversial (2–4). Although some translatome and proteome profiling studies suggest that alternative splicing contributes significantly to proteomic diversity (5, 6), a systematic analysis of over 100 published mass spectrometry (MS)-based shotgun proteomics data sets showed that most protein-coding genes have a single dominant isoform irrespective of tissue or cell type (7).

In shotgun proteomics experiments, proteins are enzymatically digested into peptides, which are subsequently fractionated and analyzed by liquid chromatography (LC)-MS/MS. The most commonly used protease in proteomics is trypsin, which cleaves at the C terminus of lysine and arginine with high efficiency and specificity (8) and produces peptides of optimal length and charge characteristics for tandem MS sequencing. A major challenge in using LC-MS/MS data for the confirmation of splice isoforms is their limited sequence coverage. Exon-exon junction spanning peptides provide direct evidence for the translation of splice isoforms and thus delineate protein isoform complexity and improve gene and isoform annotation (9–11). However, the ability to detect junction-spanning peptides in shotgun proteomics experiments is currently unknown.

We studied the proteomic coverage of exon-exon junctions in three publicly available proteomics data sets and found that trypsin preferentially cleaves exon-exon junctions and thus hinders the detection of junction-spanning peptides. This phenomenon was explained by evolutionarily conserved preferential nucleotide usage at exon boundaries according to nucleotide sequence analysis of five eukaryotic genomes. Our *in silico* and experimental analyses showed that complementary digestion schemes are essential to study the translation of alternative mRNA splicing to proteome diversity.

## MATERIALS AND METHODS

### 

#### 

##### Data sets

Three publicly available label-free shotgun proteomics data sets were used in this study, including the CPTAC_CRC data set (12), the NCI-60 cell lines data set (13), and label-free data from ProteomicsDB (14). These data sets were selected based on their high quality and large sample size. The CPTAC_CRC data set includes proteomics data from 95 colorectal cancer tumor samples, whereas the NCI-60 cell lines data set includes proteomics data from 59 cancer cell lines. We previously generated *proBAM* files for these data sets using the *proBAMsuite* (15). The *proBAM* files map peptide spectrum matches (PSMs)^1^ from proteomics studies to the genome, making it possible to analyze PSMs in the context of the genome. ProteomicsDB is a comprehensive human proteome database constructed using data from 16,857 LC-MS/MS experiments involving human tissues, cell lines, and body fluids (14). We downloaded a total of 550,904 unique proteotypic peptide sequences identified from label-free experiments in the database. Based on the genome annotation described below, we mapped these peptide sequences to the human genome using *proBAMsuite* and then generated *proBAM* files for downstream analyses.

##### Annotations of the Five Eukaryotic Genomes

We used the function *PrepareAnnotationRefseq* in the R package *customProDB* (16) to prepare genome annotation, including protein sequences and the loci of exons for all protein coding transcripts of Human (hg19), mouse (mm10) and *C. elegans* (ce10) from the UCSC table browser. The genome annotations of *S. cerevisiae* and *S. pombe* were collected from the Ensembl database (ENSEMBL FUNGI 26) using the function *PrepareAnnotationEnsembl* in the same package. Our entire data set contained 38248, 29934, 27721, 6692, and 5144 coding transcripts for human, mouse, *C. elegans, S. pombe*, and *S. cerevisiae*, respectively.

##### Peptide Number and Distance from Splice Junctions

To analyze the relationship between locations of the observed peptide termini and exon boundaries, we examined the relative distance between peptide termini and exon boundaries. The genomic locations of identified peptides within a *proBAM* file were overlapped with locations of the exons to determine: (1) how many peptides started at 0∼10nt relative to 3′ splice sites (5′ exon boundaries); (2) how many peptides terminated at 0∼10nt relative to 5′ splice sites (3′ exon boundaries); (3) how many junction peptides started 1∼10nt relative to the end of the 5′ donor exons; and (4) how many junction peptides terminated 1∼10nt relative to the start of the 3′ acceptor exons. When counting (1) and (2), we specifically excluded the first and last coding exons for each protein. The Z-test was used to determine locations that had significantly more peptides.

##### Amino Acid Composition at Splice Junctions

To examine amino acid composition at splice junctions of protein coding genes, we used the mappings between exon locations and protein sequences. We counted the terminal amino acids in each exon in the data set, including both exon-ending residues encoded by the last triplets of 5′ donor exons and junction residues encoded by triplets crossing exon-exon junctions. For each amino acid, we calculated the fold change compared with its background usage across all protein sequences in each genome data set.

To eliminate double counting of splice junctions shared between transcripts, we counted the number of unique splice junctions for each genome. We included coding transcripts only and discarded exons in UTR regions.

##### Consensus Nucleotide Sequence Pattern at Splicing Sites

We extracted nucleotide sequences at both 5′ and 3′ splice sites (10bp on exon side and 10bp on intron side) of each splice junctions in coding region (5′ or 3′ UTRs were excluded) of protein coding transcripts from genome sequence. After calculating the nucleotide composition at each position, the R package *seqLogo* was used to plot the corresponding sequence logo for sequences 4 base pairs up- and downstream from the splicing site.

##### In Silico Digestion Using Six Enzymes

Mature human protein sequences from the abovementioned Refseq annotation (downloaded from UCSC table browser) were digested with Trypsin, Chymotrypsin, Glu-C, Lys-C, Asp-N, and Arg-C, respectively, into peptides using chainsaw from Protewizard_3_0_10922 (17). Only peptides with no missed cleavage ranged between 8 to 25 amino acids were considered.

##### Shotgun Proteomics Profiling of RKO Cells

RKO cell pellets were lysed in 50% trifluoroethanol/50 mm ammonium bicarbonate (AmBic), pH 8.0 with sonication. Protein concentration was estimated using a bicinchoninic acid (BCA) assay (Pierce, Rockford, IL) and an aliquot of 250 μg total protein was used for each individual digestion with trypsin and chymotrypsin, respectively. Each peptide digest was desalted using a 1 cc, 100 mg, SEP-Pak C18 cartridge under vacuum (Waters Corporation, Milford, MA). After desalting, peptide samples were evaporated to dryness *in vacuo* and reconstituted in 10 mm triethylammonium bicarbonate (TEAB). A basis reverse phase LC fractionation was performed with an XBridge C18, 250 × 4.6 mm analytical column containing 5 μm particles and equipped with a 20 × 4.6 mm guard column (Waters, Milford, MA); flow rate was 0.5 ml/min. The mobile phases consisted of 10 mm TEAB (pH 7.5) as component A and 10 mm TEAB and 90% ACN (pH 7.5) as component B. Sample separation was accomplished using the following linear gradient: from 0 to 5% B in 10 min, from 5 to 35% B in 60 min, from 35 to 70% B in 15 min, and held at 70% B for an additional 10 min. Sixty fractions were collected during the LC separation and were concatenated into 15 fractions by combining every fourth fraction (*e.g.* 1 + 5 + 10…; 2 + 6 + 11…). Each concatenated fraction was then analyzed by liquid chromatography-tandem mass spectrometry (LC-MS/MS).

LC-MS/MS analyses were done on a Thermo Q-Exactive Plus instrument (Thermo Electron, San Jose, CA), operating in nanospray mode. Peptides were resolved on a 100 mm × 15 cm fused silica capillary column (Polymicro Technologies, LLC., Phoenix, AZ) packed with 3 μm, 120 Å ReproSil-Pur C18 (Dr. Maisch GmbH, Germany). Liquid chromatography was carried out at ambient temperature at a flow rate of 0.3 ml/min using a gradient mixture of 0.1% (v/v) formic acid in water (solvent A) and 0.1% (v/v) formic acid in ACN (solvent B). A full scan was obtained for eluting peptides in the range of 380–1800 amu followed by twelve data-dependent MS/MS scans. MS/MS spectra were recorded using dynamic exclusion of previously analyzed precursors for 20 s. MS/MS spectra were generated by higher energy collision induced dissociation (HCD) of the peptide ions at a normalized collision energy of 27% to generate a series of b- and y-ions as major fragments. Thermo RAW files are accessible through ftp://massive.ucsd.edu/MSV000081188/updates/2017-10-19_xjwang_ad93f39b/raw/.

For each data set generated using a specific enzyme, IDPicker v3.0.564(18) was used to report the peptide identifications that coupled together 15 files searched by MS-GF+ Beta (v9517) (19) and Myrimatch v2.1.132(20) against RefSeq human protein database version 54 (including 34,590 protein sequences). MyriMatch employed precursor tolerances of 15 ppm; allowed fragments to vary by up to 20 ppm, and considered semi-tryptic peptides equally with fully-tryptic peptides, allowed for isotopic error in precursor ion selection [-1, 2], conducted on-the-fly peptide sequence reversal, and applied static +57 modifications to cysteines and dynamic +16 oxidations to methionines. MyriMatch also dynamically added pyroglutamine modifications to the N termini of peptides starting with Gln residues. A minimum peptide length of 5 was required and a maximum number of 4 missed cleavages was allowed. MS-GF+ Beta employed the same precursor tolerances of 15 ppm; a fragmentation method of 3 (HCD), an instrument ID of 3 (Q-Exactive), considered semi-tryptic peptides equally with fully-tryptic peptides, allowed for isotopic error [-1, 2] in precursor ion selection, conducted on-the-fly peptide sequence reversal, and applied static +57 modifications to cysteines and dynamic +16 oxidations to methionines. MS-GF+ also dynamically added pyroglutamine modifications to the N termini of peptides starting with Gln residues. A minimum peptide length of 6 and a maximum peptide length of 40 were required. PSM tables (see supplementary File S1 for details) were generated from IDPicker report with a PSM Q value cutoff of 0.015 (ftp://massive.ucsd.edu/MSV000081188/search/Full_assembly.idpDB) and converted to *proBAM* files as described above. A minimum peptide length of 6 was required for all downstream analyses. MS/MS identification results were visualized using the proteomics data viewer (PDV, manuscript under preparation) available at http://pdv.zhang-lab.org, and annotated spectra for all junction peptides are available in supplementary File S2.

## RESULTS

We studied the genomic locations of all peptides identified in a large-scale shotgun proteomics analysis of 95 colorectal cancer (CRC) specimens published by the Clinical Proteomic Tumor Analysis Consortium (CPTAC) (12). Using *proBAMsuite,* a bioinformatics framework for genome-based representation and analysis of proteomics data, we mapped 68,876 (94.6%) out of 72,819 distinct peptide sequences identified in the CPTAC_CRC data set to unique genomic locations (15). Among the uniquely mapped peptides, 16,614 (24.1%) were exon-exon junction peptides that provide direct evidence for RNA splicing (15). Surprisingly, 6.7% (*n* = 3,476) of the peptide sequences uniquely mapped within exons either started or terminated at the exon boundaries. This is almost twice the expected rate of 3.8% estimated based on a medium peptide length of 13 amino acids in this data set and a medium length of 66 amino acids for exons (2/(66–13)). Similarly, analysis of another large-scale shotgun proteomics data set of the NCI-60 cell lines (13) showed that 5.7% of the peptide sequences within exons either started or terminated at the exon boundaries. To further test this observation in noncancer settings, we used a collection of 550,904 proteotypic peptide sequences from the comprehensive, LC-MS/MS data-derived human proteome database, ProteomicsDB (14). We mapped 546,939 (99.3%) of these peptide sequences to human genome based on the RefSeq annotation. Among the 523,086 (95.6%) uniquely mapped peptide sequences, 128,346 (24.5%) were exon spanning. Consistent with our observations in the cancer data sets, 5.6% (*n* = 22,290) of the peptide sequences uniquely mapped within exons either started or terminated at the exon boundaries.

**Fig. 1.**
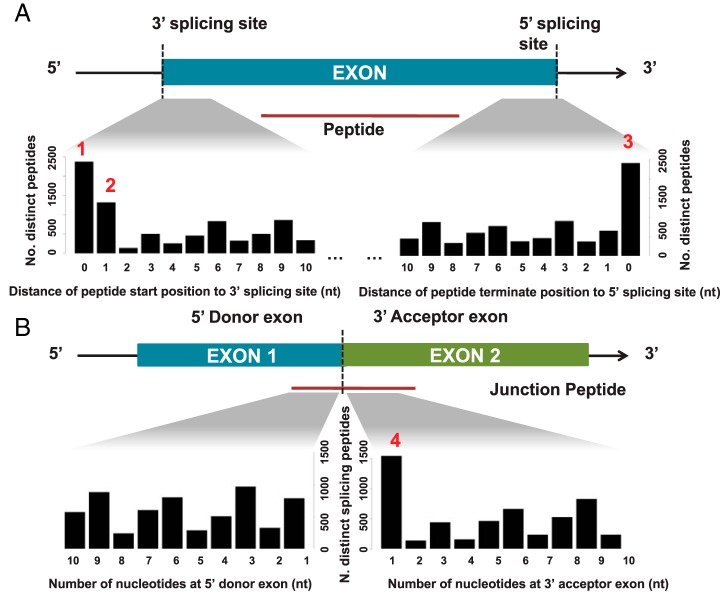
**Enrichment of tryptic peptide termini proximal to exon boundaries.** Distribution of the relative distance between the locations of the observed peptide termini in the CPTAC_CRC data set and exon boundaries is plotted for (*A*) all distinct peptides or (*B*) exon-exon junction peptides. Only peptides that terminate within 10 base pairs of a splicing site were counted. An enrichment of tryptic peptide termini proximal to exon boundaries was observed, most remarkably for the four positions labeled 1–4 in red.

To further understand the relationship between locations of the observed peptide termini and exon boundaries, we examined the relative distance between peptide termini and exon boundaries in the CPTAC_CRC data set. Fig. 1*A* shows the relative distance distribution for all observed distinct peptides that started or terminated within 10 base pairs of a splice site. A more focused analysis was also performed for all distinct exon-exon junction peptides (Fig. 1*B*). Interestingly, we observed an enrichment of peptide termini proximal to exon boundaries, most remarkably for peptides that either (1) started at a splice site (*p* = *0.010*, Z-test), (2) started 1bp downstream of a 3′ splice site (*p* = 0.031), (3) terminated at a splice site (*p* < 0.005), or (4) terminated 1bp downstream of a 3′ splicing site (*p* = 0.016). We refer hereafter to these as type 1 through 4 peptides, respectively (Fig. 1). These four types of peptides accounted for around 7% of the uniquely mapped peptides. A similar pattern was observed in the NCI-60 cell lines data set and the proteotypic peptide sequences from ProteomicsDB (supplemental Fig. S1).

**Fig. 2.**
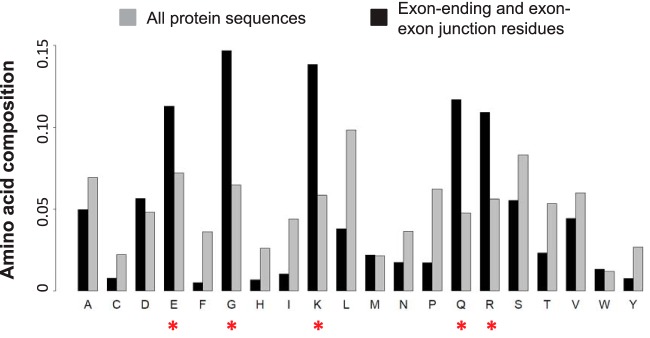
**Enrichment of certain amino acids among exon-ending and exon-exon junction residues.** Gray bars depict overall amino acid composition for all human protein sequences, whereas black bars depict amino acid composition for all exon-ending and exon-exon junction residues. Five amino acids indicated by red asterisks were enriched more than 1.5 fold among the exon-ending and junction residues, including glutamine (Q, 2.45 fold), lysine (K, 2.37 fold), glycine (G, 2.26 fold), arginine (R, 1.94 fold), and glutamic acid (E, 1.56 fold).

To explain the observed enrichment of type 1∼4 peptides, we calculated the amino acid composition of the terminal residues encoded by all exons of RefSeq human coding transcripts, including exon-ending residues encoded by the terminal triplets of 5′ donor exons and junction residues encoded by triplets crossing exon-exon junctions. Compared with the overall amino acid composition for all human coding transcripts, five amino acids were enriched more than 1.5 fold among the exon-ending and junction residues, including glutamine (Q, 2.45 fold), lysine (K, 2.37 fold), glycine (G, 2.26 fold), arginine (R, 1.94 fold), and glutamic acid (E, 1.56 fold) (Fig. 2). These five most enriched amino acids accounted for 63% of all exon-ending and junction residues, compared with the background rate of 30%. Two of the five amino acids, lysine and arginine, occurred in 25% of the exon-ending and junction residues. Because trypsin cleaves peptides on the carboxyl side of lysine and arginine, their enrichment among exon-ending and junction residues explains the enrichment of type 1∼4 peptides in shotgun proteomics studies using trypsin for protein digestion (Fig. 1).

Because the same splice junction could be counted multiple times if it was shared by multiple isoforms, we further calculated the number of unique exon-exon junctions in the genome and the number of unique junctions with lysine or arginine at exon-ending or exon-exon junction positions, referred to hereafter as “K/R junctions.” As shown in supplemental Table S1, K/R junctions constituted 24.9% of all unique junctions in the human coding genome.

**Fig. 3.**
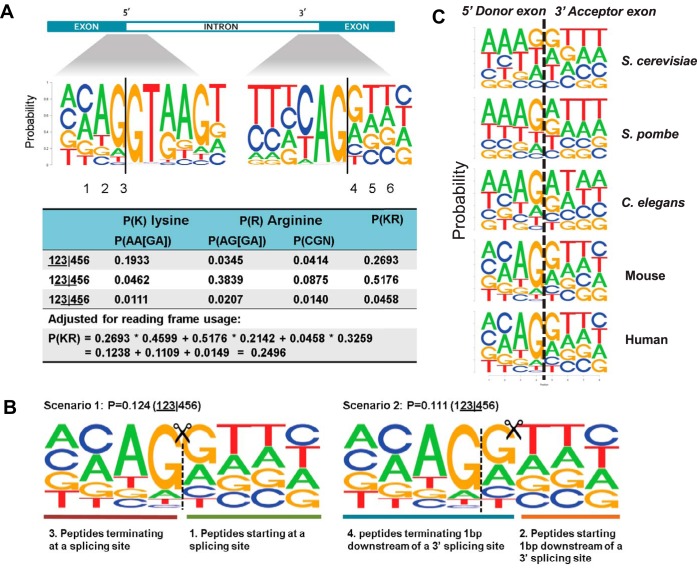
**Preferential cleavage by trypsin at splice sites.** *A*, Consensus sequences on either side of the exon-intron boundaries for all coding genes in the human genome. The first three rows in the table list the occurrence probabilities of lysine (K) and arginine (R) at splice sites for each of the three reading frames, respectively. As shown in the last row of the table, adjusted for reading frame usage, one quarter of the splicing sites can be recognized by trypsin as potential cleavage sites, which is twice the genome-wide average (11%). *B*, Two scenarios resulting in the type 1–4 peptides labeled in (a). The vertical dashed lines represent the splicing sites, and the scissors indicate the trypsin-digesting sites. The occurrence probability of scenario 1 is 0.124, in which the digestion produces type 1 and 3 peptides. The occurrence probability of scenario 2 is 0.111, in which type 2 and 4 peptides are generated. *C*, Consensus sequences on the exon side of five eukaryotic genomes. There is no significant difference between the human and mouse 5′ donor exon. The “AG-R” pattern seems to become more pronounced during evolution.

Amino acid bias is determined by nucleotide usage and exon phases. To further understand the enrichment of K/R junctions, we calculated the consensus nucleotide sequences on either side of the exon-intron boundaries for all coding genes in the human genome. We observed an AG-R (G/A) pattern at the exon boundaries, adjacent to the canonical GU-AG motif at the intron side (Fig. 3*A*). Adjusted for reading frame usage, we estimated that 25% of all triplets involving the terminal nucleotide residue of an exon code for lysine or arginine, which is more than twice the genome-wide average of 11.4% (Fig. 3*A*). The four peptide types described above can be generated in two distinct scenarios determined by the position of the splice junction within a codon, where each scenario produces two types of peptides (Fig. 3*B*). In scenario 1, the trypsin digestion site is located exactly at the splicing site, thus creating type 3 and type 1 peptides. In scenario 2, trypsin cleaves at 1bp downstream of the splicing site, thus creating type 4 and type 2 peptides.

We investigated the nucleotide constraints at exon boundaries in four other eukaryotic genomes and found a similar pattern. As shown in Fig. 3*C*, the “AG-R” pattern seems to become more pronounced through evolution. We found comparable K/R junction ratios among these four species, except for the yeast *Saccharomyces cerevisiae* (supplemental Table S1), which is likely because of the lack of introns in most *Saccharomyces cerevisiae* genes. The results indicate that in addition to human studies, trypsin digestion may also lead to enriched observation of type 1∼4 peptides in shotgun proteomics analyses of other species.

Preferential generation of type 1∼4 peptides by trypsin digestion reduces the chance of verifying splice junctions by MS-based proteomics. In the CPTAC_CRC data set, 4579 uniquely mapped peptide sequences fell into type 1∼3 categories and thus could not be used to verify splice junctions. Another 1148 uniquely mapped peptide sequences were type 4 peptides, which have limited value in splice junction verification, because they cover only a single, relatively constrained nucleotide on the 3′ acceptor exon (supplemental Fig. S2).

We then performed *in silico* digestion to evaluate the impact of enzyme selection on splice junction detectability. After *in silico* digestion, 860260, 881509, 709022, 567118, 836211, and 568171 peptides were obtained for Trypsin, Chymotrypsin, Glu-C, Lys-C, Asp-N and Arg-C, respectively. Resulted peptides were stored in *proBAM* files, which were used to calculate whole coding genome, junction, and K/R junction coverages for each enzyme (Table I). Trypsin, Chymotrypsin, and Asp-N digestions showed the highest coding DNA sequence (CDS) coverages, close to half of all CDS of the human genome.

**Table I:** Proteome coverage, number of detectable junctions, and K/R junctions from in-silico digestion of six different proteases. Peptide length was specified to be within 8 to 25 residues. The calculation was based on peptides uniquely mapped to genome. Type 4 peptides were not included in the study because of mapping uncertainty introduced by the single nucleotide coverage on the 3' acceptor exon

	Cleavage site	Proteome coverage	#Detectable junctions (coverage)	#Detectable K/R junctions (coverage)
Trypsin	C-terminal of R and K	46.9%	70,726 (38.8%)	1,454 (3.2%)
Lys-C	C-terminal of K	28.9%	47,954 (26.3%)	6,787 (14.9%)
Glu-C	C-terminal of D	37.8%	66,367 (36.4%)	18,467 (40.5%)
Chymotrypsin	C-terminal of F, Y, L, W and M	46.8%	89,371 (49.0%)	22,564 (49.5%)
Asp-N	N-terminal of D	45.3%	82,761 (45.4%)	20,897 (45.9%)
Arg-C	C-terminal of R	29.5%	46,339 (25.4%)	7,850 (17.2%)

**Fig. 4.**
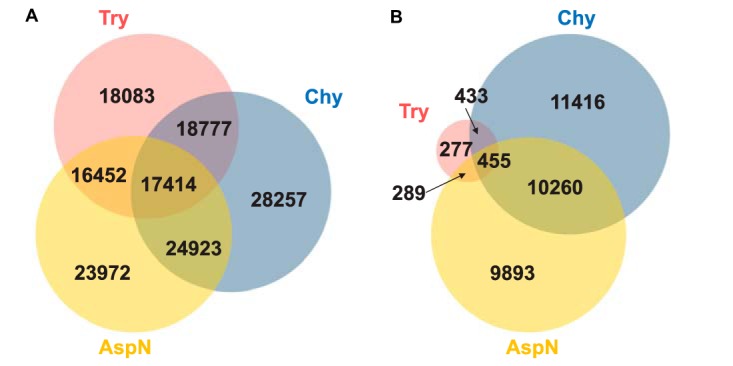
**Venn-diagram showing shared and unique junctions (*A*) and K/R junctions (*B*) with peptide evidence in *in-silico* digestion experiments using trypsin, chymotrypsin and Asp-N.** Peptide length was specified to be between 8 and 25 residues.

However, trypsin digestion resulted in extremely low coverage (3.2%) for K/R junctions, and the overall junction coverage also dropped to 38.8% compared with the 46.9% proteome coverage (Table I). We note that the detectable K/R junctions were present because of Proline followed by Lysine or Arginine. Lys-C and Arg-C digestions also produced reduced K/R coverage because these enzymes digest after Lysine (Lys-C) and Arginine (Arg-C), respectively. For chymotrypsin and Asp-N, the coverage ratios for junction and K/R junction were similar to those for the whole coding DNA sequence (CDS). A 2% increase was found for chymotrypsin (Table I). Fig. 4 depicts the overlap of detectable junctions and K/R junctions between the three top performing enzymes: Trypsin, Chymotrypsin and Asp-N. As shown in the figure, each enzyme digestion yielded a unique inventory of detectable junction peptides, and their combination would produce a much more comprehensive inventory. Together, the six enzymes created a total of 161,125 detectable junctions, and only 1029 were common across all enzyme digestions (supplemental Fig. S3). Chymotrypsin digestion provided the largest number of detectable junctions and K/R junctions (Table I).

**Fig. 5.**
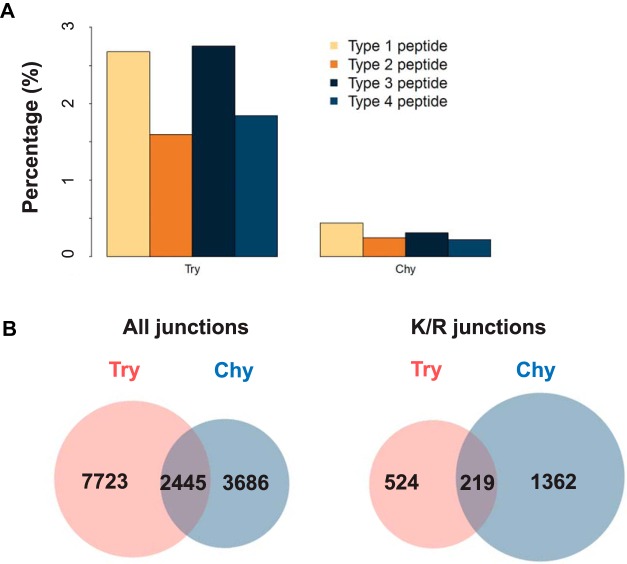
**Impact of protein digestion enzyme selection on splice junction verification.** *A*, Digestion of RKO protein lysate using trypsin (Try) resulted in a higher proportion of type 1–4 peptides compared with using chymotrypsin (Chy). See Fig. 3*B* for the graphical representation of the different types of peptides. *B*, Comparison of the numbers of junctions with supporting peptides identified in trypsin or chymotrypsin digested samples. The left Venn diagram includes all junctions, whereas the right Venn diagram includes only junctions with lysine (K) or arginine (R) at the junction site and which cannot be identified in tryptic digests.

We experimentally tested a complementary digestion scheme by performing parallel shotgun proteomics experiments on RKO cell proteomes using trypsin and chymotrypsin, respectively. In contrast to trypsin, chymotrypsin cleaves at the carboxyl side of phenylalanine (F), tryptophan (W), tyrosine (Y), and leucine (L), which are mostly under-represented among exon-ending and exon-exon junction residues (Fig. 2). As expected, trypsin digestion led to a much higher number of peptide identifications. The number of identified distinct peptides with trypsin digestion was 90,710 (61,829 peptide sequences), whereas chymotrypsin digestion identified 41,874 (32,465 peptide sequences) (supplemental Table S2). However, consistent with our *in silico* analysis, chymotrypsin digestion generated a much lower proportion of type 1∼4 peptides than trypsin digestion (Fig. 5*A*), with the summed ratios being 8.9% and 1.2% for trypsin and chymotrypsin, respectively.

We further calculated the numbers of junctions covered by proteomic data in the two experiments. For each experiment, we limited our comparisons to the uniquely mapped junction peptides. Type 4 peptides were not included in the study because of mapping uncertainty introduced by the single nucleotide coverage on the 3′ acceptor exon (supplemental Fig. S2). Junction peptides with only two nucleotide coverage on the 3′ acceptor exon were also excluded, although the number of such cases was very small (Fig. 3*A*). We found 11,783 and 8036 uniquely mapped junction peptide sequences in the trypsin experiment and the chymotrypsin experiment, respectively, corresponding to 10,168 and 6131 verified junctions, respectively (Fig. 5*B*). The numbers of detected K/R junctions were 743 and 1581 for trypsin and chymotrypsin, respectively (Fig. 5*B*). All of the 743 K/R junctions detected in the trypsin experiment resulted from miscleaved peptides or proline followed by lysine or arginine. Of the 3686 junctions that were detected in the chymotrypsin experiments, but not the trypsin experiment, 1362 (37%) could not have been detected with completely cleaved tryptic peptides because of the presence of lysine or arginine at splicing sites (Fig. 5*B*). Thus, experimental data support the *in silico* calculation and confirm the preferential cleavage by trypsin at splicing sites, a previously unknown limitation that may hinder understanding of protein isoform complexity.

**Fig. 6.**
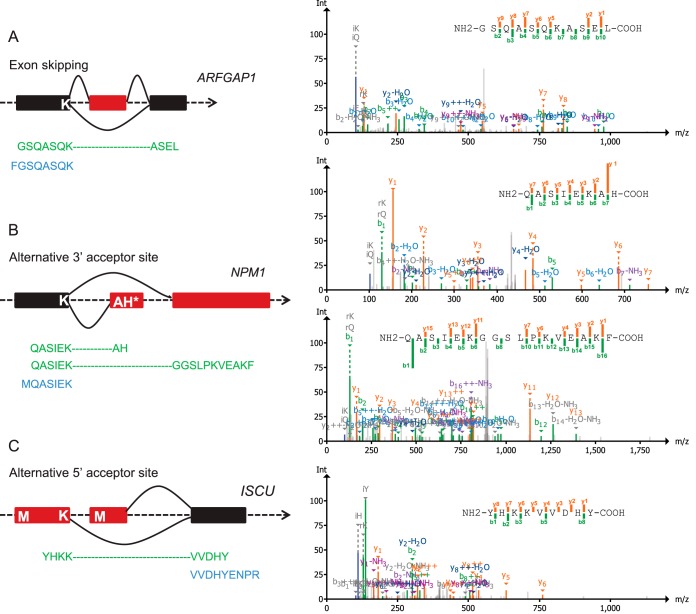
**Examples showing the effect of trypsin preferential cleavage at junction site on protein isoform identification in the RKO data.** Peptides from chymotrypsin digestion are colored in green and those from trypsin are in blue. Boxes in red represent the alternative exons. *A*, An illustration of exon skipping events in ARFGAP1 gene. *B*, An illustration of alternative 3′ acceptor site in the NPM1 gene. The isoform 3 utilizes an alternate 3′ terminal exon, resulting in a shorter protein with distinct C terminus compared with isoform 1/2. *C*, An illustration of an alternative 5′ acceptor site in the ISCU gene. Representative spectra for the 4 green-colored peptide sequences from chymotrypsin digestion are shown on the right.

We use three examples from the RKO data to further illustrate such limitation. As shown in Fig. 6*A*, an exon skipping event in gene ARFGAP1(ADP-ribosylation factor GTPase activating protein 1) was confirmed by a peptide identified from the chymotrypsin digestion, but data from trypsin digestion failed to provide evidence for this event because of the presence of lysine at the junction site. Fig. 6*B* shows that K/R junction could confound the proteomic confirmation of isoforms resulted from alternative 3′ accepter site. Nucleophosmin (NPM1) has three isoforms, isoform 3 utilizes an alternate 3′-terminal exon, compared with isoform 1/2, resulting in a shorter protein (isoform 3) with a distinct C terminus. Notably the 3′-terminal exon of isoform 3 contains only 2 amino acids (Fig. 6*B*), thereby it is impossible to find mass spectral evidence for isoform 3 if the protein is cleaved by trypsin at the immediate upstream junction lysine. Similarly, a peptide from chymotrypsin digestion confirmed the usage of an alternative 5′ acceptor site in gene ISCU (iron-sulfur cluster assembly enzyme), whereas the peptide from trypsin digestion failed to provide evidence on alternative site usage (Fig. 6*C*).

## DISCUSSION

We documented the preferential cleavage at exon-exon junction sites by trypsin digestion and revealed evolutionarily conserved preferential nucleotide usage at exon boundaries. Although the nucleotide constraints of the exon boundaries have been known for some time (21), few studies have investigated the effect on amino acid composition of the encoded proteins (22–24). Because trypsin is the most commonly used enzyme in proteomics experiments, our finding has significant implications in the design of proteomics experiments, particularly when the objective is to investigate the translation of alternative splicing events to the proteome level.

The contribution of alternative splicing to proteome diversity is an active area of research, and there is conflicting evidence as to how much the transcript diversity can be translated to proteins (5–7). The nearly exclusive use of trypsin as the protein digestion enzyme in shotgun proteomic analyses hinders the detection of junction-spanning peptides and makes it difficult to delineate protein isoform complexity. This may partially explain the conflicting reports. Trypsin digestion may also contribute to the limited success in identifying novel splicing peptides in recent proteogenomics studies (16, 25, 26). Moreover, ongoing efforts that optimize digestion procedures to enrich fully tryptic peptides (27) will further reduce the chance of verifying exon-exon junctions in trypsin digested samples. Indeed, an analysis of mouse exon-exon junctions that could be confirmed by shotgun proteomics in trypsin digested samples showed that the detection ratio would drop from 70.9% to 46% if mis-cleaved peptides were removed (10).

The use of alternative protein-digesting enzymes with orthogonal specificity to trypsin, such as chymotrypsin, will not only generate different peptide sequences that complement those identified based on trypsin digestion, but will also provide peptide evidence for junctions that are impossible to identify in tryptic digests. Complementary digestion schemes will enable more comprehensive study of how differential RNA splicing drives flexible proteome adaptation in distinct biological states.

## DATA AVAILABILITY

The shotgun proteomics data of RKO cells have been deposited in the public proteomics repository MassIVE and are accessible at ftp://massive.ucsd.edu/MSV000081188, including the Thermo RAW files, IDPicker report, annotated spectra for all junction peptides, and other relevant data.

## Supplementary Material

Supplemental Data

## Footnotes

^1^ The abbreviations used are:

PSM(s): peptide spectrum match(es)
CDS: coding DNA sequence
ProBAM: proteomics BAM
IGV: integrative genomics viewer
CPTAC: Clinical Proteomic Tumor Analysis Consortium.
