# Supplementary material for: Detection of Proteome Diversity Resulted from Alternative Splicing is Limited by Trypsin Cleavage Specificity
Source: Mol Cell Proteomics. 2017 Dec 8;17(3):422–30. doi: 10.1074/mcp.RA117.000155 (PMC5836368; doi:10.1074/mcp.RA117.000155)
Supplement: Supplemental Data [file supp_17_3_422__index.html]

Detection of proteome diversity resulted from alternative splicing is limited by trypsin cleavage specificity — Trypsin preferentially cleaves exon-exon junctions — Detection of Proteome Diversity Resulted from Alternative Splicing is Limited by Trypsin Cleavage Specificity — Trypsin Preferentially Cleaves Exon-Exon Junctions — Supplemental Data 

# Detection of Proteome Diversity Resulted from Alternative Splicing is Limited by Trypsin Cleavage Specificity

## Supplemental Data

- Supplementary Figures and Tables - Supplementary Figures 1-3 Supplementary Tables 1-2
- Supplementary File 1 - All identified PSMs for RKO protein lysate digested with trypsin and chymotrypsin.
