# Supplementary material for: Detection of Proteome Diversity Resulted from Alternative Splicing is Limited by Trypsin Cleavage Specificity
Source: Mol Cell Proteomics. 2017 Dec 8;17(3):422–30. doi: 10.1074/mcp.RA117.000155 (PMC5836368; doi:10.1074/mcp.RA117.000155)
Supplement: Supplemental Data [file supp_RA117.000155_4751_1_supp_20079_1z0r6j.pdf]

Detection of proteome diversity resulted from alternative splicing is limited by trypsin cleavage specificity

Xiaojing Wang[1,2], Simona G. Codreanu[3], Bo Wen[1,2], Kai Li[4], Matthew C. Chambers[3], Daniel C. Liebler[3,5], Bing Zhang*[1,2]

[1]Lester and Sue Smith Breast Center, Baylor College of Medicine, Houston, Texas 77030, USA. [2]Department of Molecular and Human Genetics, Baylor College of Medicine, Houston, Texas 77030, USA. [3]Department of Biochemistry, Vanderbilt University School of Medicine, Nashville, Tennessee 37232, USA. [4]BGI-Shenzhen, Shenzhen, Guangdong 518083, China. [5]Jim Ayers Institute for Precancer Detection and Diagnosis, Vanderbilt-Ingram Cancer Center, Nashville, Tennessee 37232, USA

* Correspondence should be addressed to B.Z. (bing.zhang@bcm.edu).

SUPPLEMENTARY INFORMATION

- **Supplementary Figure 1. Enrichment of tryptic peptide termini proximal to exon boundaries in the NCI-60 cell line data set and the proteotypic peptides from ProteomicsDB.**
- **Supplementary Figure 2. An example demonstrating type 4 peptide mapping ambiguity.**
- **Supplementary Figure 3. Venn-diagram showing shared and unique junctions (a) and K/R junctions (b) with peptide evidence in *in-silico* digestion experiments using six different proteases.**
- **Supplementary Table 1. The numbers of unique junctions and K/R junctions in five eukaryotic genomes.**
- **Supplementary Table 2. Peptide identification results for RKO protein lysate digested with two different enzymes.**
- **Supplementary File 1. All identified PSMs for RKO protein lysate digested with trypsin and chymotrypsin.**
- **Supplementary File 2. Annotated representative spectra for all junction peptides of RKO.**

Supplementary Figure 1. Enrichment of tryptic peptide termini proximal to exon boundaries in the NCI-60 cell line data set and the proteotypic peptides from ProteomicsDB. a. Distribution of the relative distance between the locations of the observed peptide termini in the NCI-60 cell line data set and exon boundaries is plotted for all distinct peptides (top panel) or exon-exon junction peptides (bottom panel). Only peptides that terminate within 10 base pairs of a splicing site were counted. An enrichment of tryptic peptide termini proximal to exon boundaries was observed, most remarkably for the positions labeled 1-4 in red. **b.** A similar pattern was observed in proteotypic peptides from ProteomicsDB.

a.

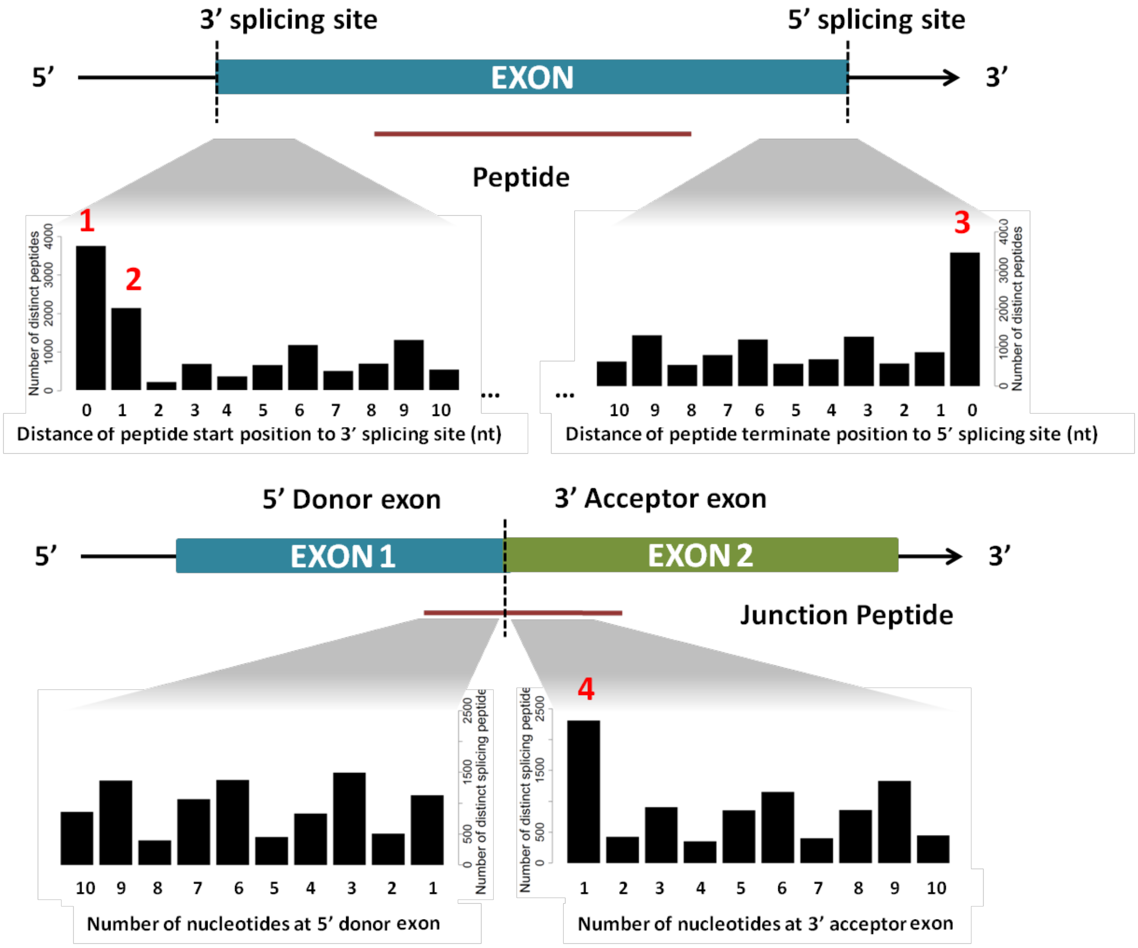

b.

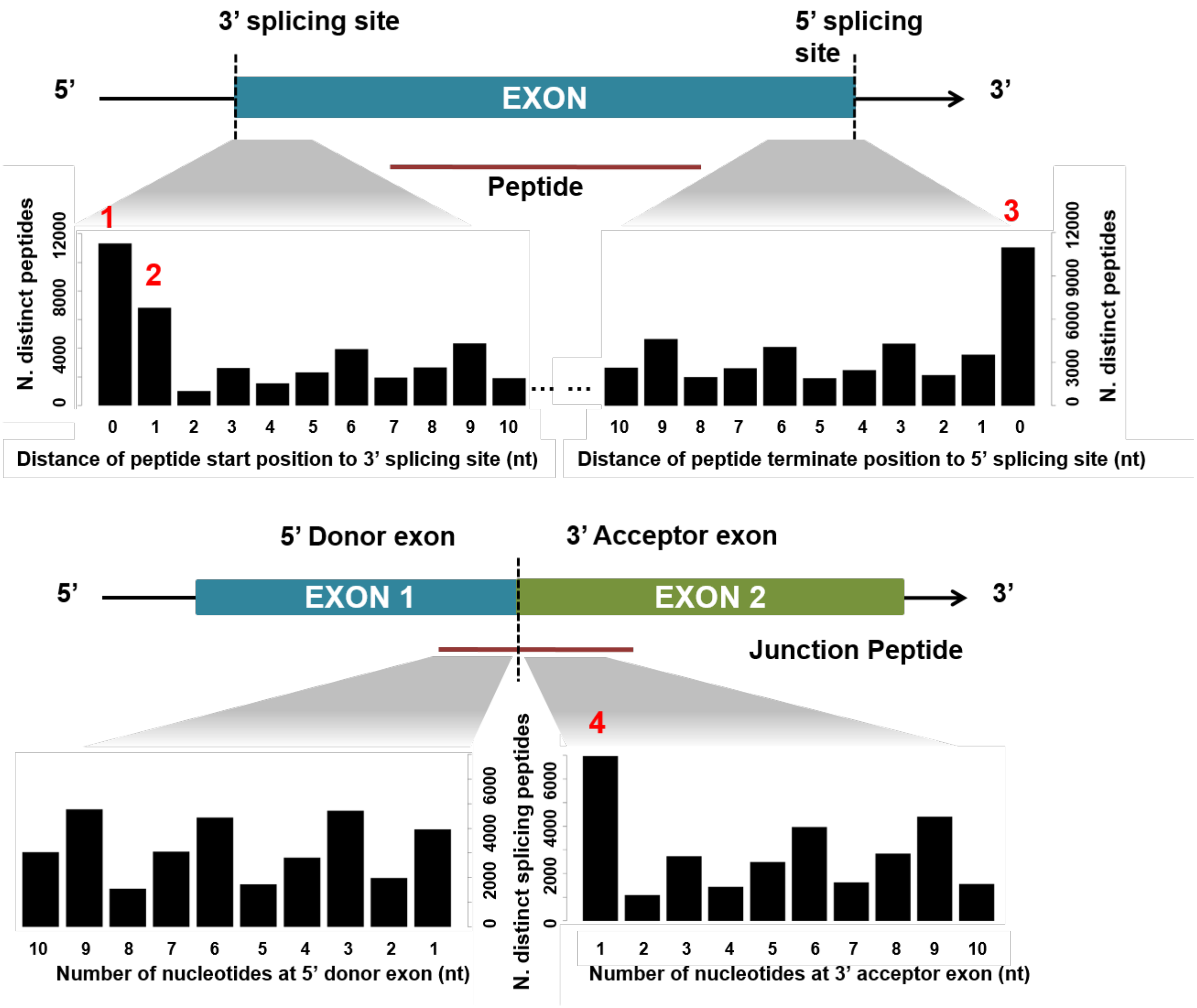
3' splicing site
5' splicing site
5'
EXON
3'
Peptide
1
2
3
N. distinct peptides
0 4000 8000 12000
0 3000 6000 9000 12000
0 1 2 3 4 5 6 7 8 9 10
10 9 8 7 6 5 4 3 2 1 0
Distance of peptide start position to 3' splicing site (nt)
Distance of peptide terminate position to 5' splicing site (nt)
5' Donor exon
3' Acceptor exon
EXON 1
EXON 2
Junction Peptide
4
N. distinct splicing peptides
0 2000 4000 6000
10 9 8 7 6 5 4 3 2 1
1 2 3 4 5 6 7 8 9 10
Number of nucleotides at 5' donor exon (nt)
Number of nucleotides at 3' acceptor exon (nt)

Supplementary Figure 2. An example demonstrating type 4 peptide mapping ambiguity. A peptide ‘IGGVQQDTILAEGLHFR’ in NCI-60 data set mapped to multiple locations (green boxes) of gene PHB2, corresponding to three splice forms (red boxes). Because of the preferential cleavage by trypsin at the splicing site + 1bp position, type 4 peptides only cover one nucleotide on the second exon and thus are difficult to associate with a unique splice junction.

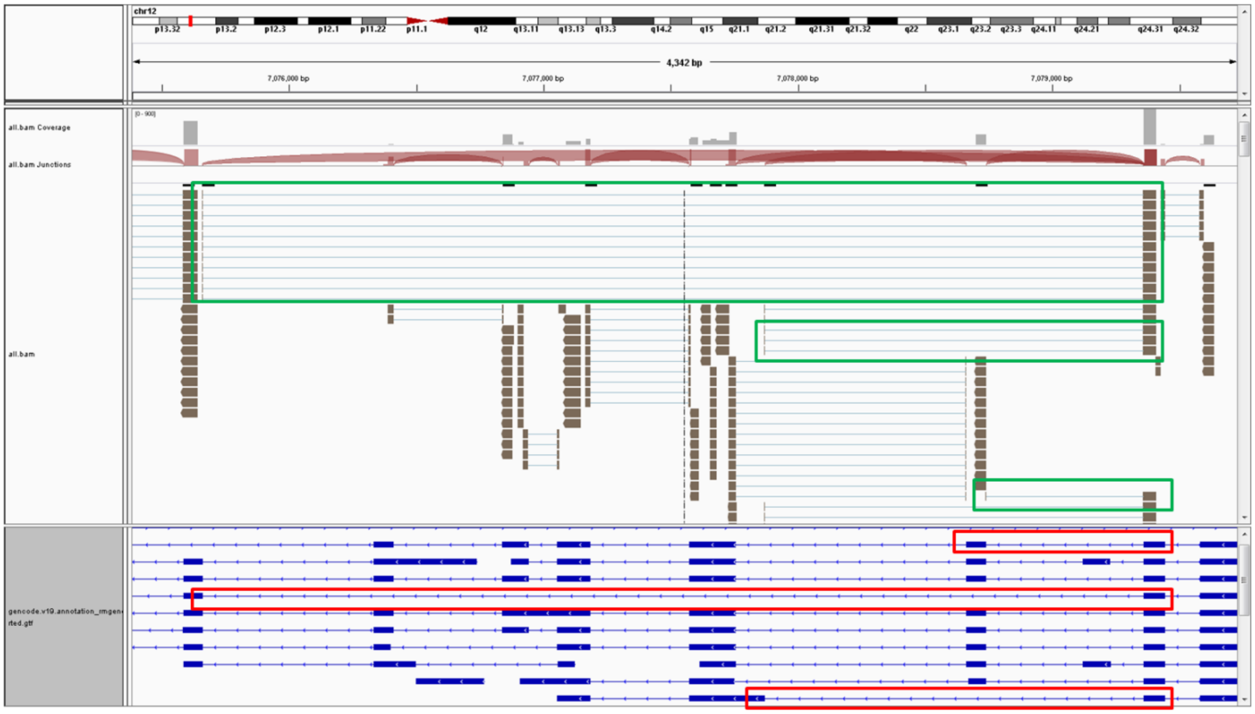

Supplementary Figure 3. Venn-diagram showing shared and unique junctions (a) and K/R junctions (b) with peptide evidence in *in-silico* digestion experiments using six different proteases. Peptide length was specified to be between 8 and 25 residues.

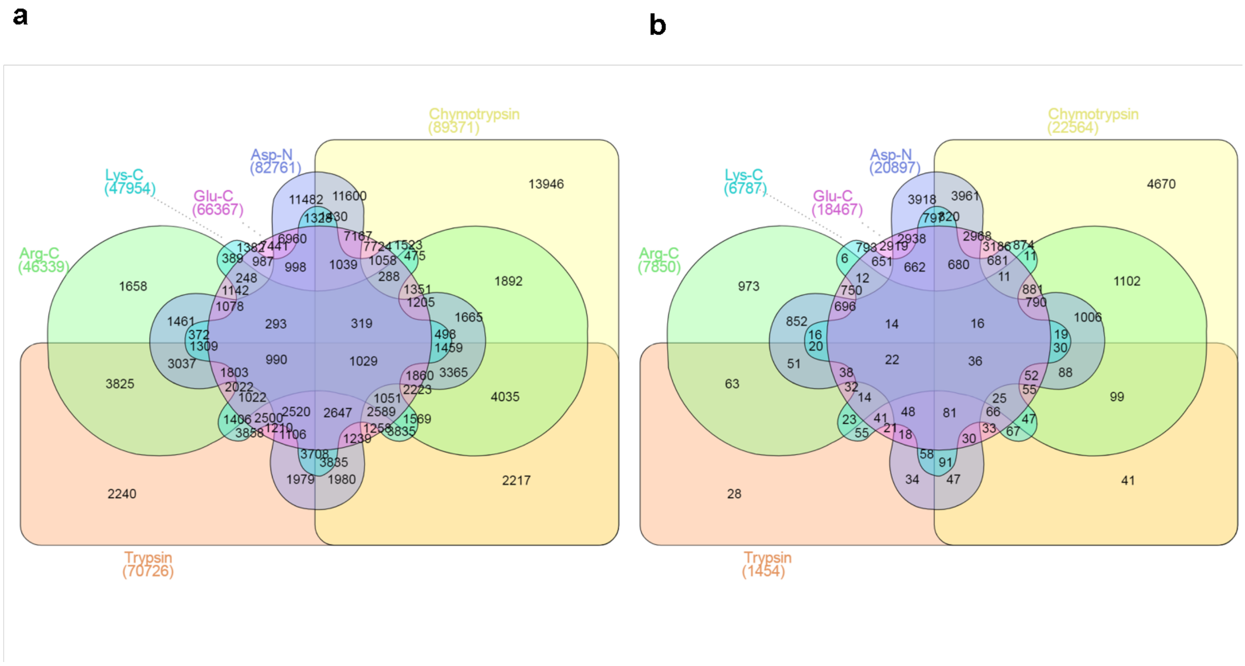

Supplementary Table 1. The numbers of unique junctions and K/R junctions in five eukaryotic genomes. The annotations used for calculation were described in **Methods**. Unique junctions were defined based on their genomic locations. The '#Unique junction aa' column indicates the total number of unique junctions encoding amino acid. Since a few splice junctions were involved in different reading frame and coding different amino acids, the numbers in column '#Unique junction aa' sometimes are slightly higher than those in column '#Unique junctions'. The ratios in column '#K/R junctions' were calculated by dividing the numbers in column '#Unique junction aa'.

	**#Unique junctions**	**#Unique junction aa**	**#K/R junctions**
**Human**	182443	183011	45561 (24.9%)
**Mouse**	176342	176626	44062 (24.9%)
C.elegans	107584	108312	25764 (23.9%)
S.pombe	5065	5065	952 (18.8%)
S.cer	342	342	52 (15.2%)

Supplementary Table 2. Peptide identification results for RKO protein lysate digested with two different enzymes.

*The number of junction peptides was only counted for uniquely mapped peptide sequence.

	#Identifiable spectra	#Distinct peptides	#Distinct peptide sequences	#Uniquely mapped peptides	#Junction peptides*
**TRY**	151344	90710	61829	57849	12921
**CHY**	63259	41874	32465	30136	8136
